# Nutritional and Antioxidant Comparison of Oil Press Cakes and Wheat Flours

**DOI:** 10.3390/molecules30244781

**Published:** 2025-12-15

**Authors:** Olina Dudasova Petrovicova, Nevena Dabetic, Milica Zrnic Ciric, Brizita Djordjevic, Vanja Todorovic

**Affiliations:** Department of Bromatology, Faculty of Pharmacy, University of Belgrade, Vojvode Stepe 450, 11221 Belgrade, Serbia; olinad@pharmacy.bg.ac.rs (O.D.P.); nevenad@pharmacy.bg.ac.rs (N.D.); milica.zrnic@pharmacy.bg.ac.rs (M.Z.C.); brizita.djordjevic@pharmacy.bg.ac.rs (B.D.)

**Keywords:** oil production byproduct, wheat flours, pumpkin, sunflower, apricot, minerals, amino acids, antioxidant activity

## Abstract

Plants are sources of compounds with important effects on health, but plant-based food industry generates substantial waste amounts, especially in oil production. This study aimed to characterize flours derived from oilseed by-products, pumpkin, sunflower, and apricot seed residues, and compare them with conventional grain flours (white and whole wheat). Nutritional composition was analyzed with emphasis on amino acid profiles performed by ion chromatography. Mineral profiles were determined by ICP-MS. Total phenolics and antioxidant activity were assessed using in vitro colorimetric microassays. Oil press cake flours showed significantly higher levels of protein and fiber compared to wheat flours (*p* < 0.05), while the latter contained more carbohydrates. Among the examined flours, pumpkin and apricot seed flours stood out with the highest potassium, while sunflower seed flour led in calcium content. Despite higher polyphenol content in wheat flours, apricot seed flour exhibited the greatest antioxidant activity, likely due to its diverse profile of hydrophilic and lipophilic bioactive compounds. These findings highlight oil press cakes as nutritionally valuable ingredients for protein-enriched and other innovative food products, aligning with circular economy principles and promoting resource efficiency in the agri-food sector.

## 1. Introduction

Animal-derived proteins are recognized for their complete amino acid profiles and high biological value, but growing awareness of environmental sustainability has led to a shift in consumer preference toward plant-based protein sources [1]. Namely, food production, particularly meat, accounts for a substantial share of anthropogenic greenhouse gas emissions [2,3]. Plant-based proteins, found in legumes, nuts, seeds, and whole grains, are environmentally favorable and promote sustainable food systems.

In addition to these primary protein sources, the residues remaining after plant oil production are also rich in proteins, fiber, and valuable bioactive compounds, yet are often discarded or used for animal feed and composting, posing environmental burdens. These materials also offer considerable potential for bioenergy production, providing an additional route for waste reduction [4]. One sustainable strategy involves valorizing such by-products by incorporating them into flours alongside conventional cereal flours, which is feasible only when the by-products meet necessary quality requirements. Residues from the oil production of pumpkin, sunflower, and apricot have gained attention as promising raw materials for baked goods [5,6,7]. The amount of these by-products generated annually on a global scale is substantial. Sunflower oil cake represents the largest portion, with up to one million tons of residues [8], whereas the by-products from pumpkin and apricot seeds are considerably smaller, amounting to tens of thousands of tons. While refined wheat flour remains the dominant choice in both household and industrial baking, it is low in essential macronutrients [9]. In contrast, oil press cake flours are naturally richer in protein, fiber, and unsaturated fatty acids, making them attractive candidates for nutritional enhancement. Beyond their macronutrient advantages, these flours are rich in essential minerals [10] and bioactive compounds, including polyphenols and carotenoids, and exhibit high antioxidant activity [11,12]. Therefore, incorporating them into conventional wheat flour could potentially yield baked goods with improved functional quality [13].

To obtain such nutritionally and functionally valuable flours, the choice of processing method plays a crucial role. Cold pressing represents a more energy-efficient and environmentally friendly oil extraction technique, preserving the natural aroma, flavor, and color of press cakes and yielding high-quality flours and oils with superior sensory properties. As a mechanical process conducted at temperatures below 50 °C, it minimizes heat exposure and thus retains sensitive nutrients that are often degraded during conventional hot-pressing or solvent extraction [14].

Despite increasing interest in circular economy approaches and the use of cold-pressed oilseed by-products, comprehensive characterization of these flours in direct comparison with conventional wheat flours remains limited. This study therefore evaluates pumpkin, sunflower, and apricot seed cake flours alongside wholegrain and white wheat flours with respect to macronutrient composition, amino acid and mineral profiles, polyphenol content, and antioxidant potential. By addressing this gap, this work provides compositional evidence for the nutritional advantages of oil press cake flours and their potential as value-added ingredients that can be reintegrated into sustainable cereal-based food systems.

## 2. Results and Discussion

### 2.1. Energy and Macronutrient Composition of Flours

The nutritional composition of five selected types of flour, pumpkin seed, sunflower seed, apricot seed, whole grain wheat, and white wheat, is presented in Table 1. The energy values of these flours ranged from 1439 kJ for pumpkin seed flour to 1522 kJ for white wheat flour.

Statistically significant differences in protein content were observed among the samples (*p* < 0.05, ANOVA), except between the wheat-based flours, which showed no significant difference (*p* > 0.05, Student’s test). Pumpkin seed flour demonstrated the highest protein content (65.2%). These results are consistent with those reported by Miedzianka et al. [15]. Sunflower (34.8%) and apricot seed flour (30.3%) showed notably higher protein levels than whole grain (10.6%) and white wheat flour (9.4%). Our findings substantiate the considerable protein content of oil press by-products and are consistent with the observations of Nemś et al. [16], who highlighted the importance of plant-based protein preparations for enhancing the nutritional quality of baked products.

Statistically significant differences in carbohydrate content were observed among all flour samples (*p* < 0.05, ANOVA). White wheat flour exhibited the highest carbohydrate content (78.2%), followed by whole grain wheat flour (70.5%). In contrast, oil press cake flours contained substantially lower carbohydrate levels: apricot (36.1%), sunflower (22.9%), and pumpkin (6.8%). These differences are consistent with the typical macronutrient composition of grains, which are naturally higher in carbohydrates compared to oilseeds. The reduced carbohydrate content of oil press cake flours suggests their potential suitability for low-carbohydrate or high-protein dietary formulations. These findings align with those reported by Hughes et al. [9], who analyzed commercial supermarket flours and observed similar distributions, particularly in carbohydrate and protein content.

Statistically significant differences in fat content were observed among the samples (*p* < 0.05, ANOVA), except between the wheat-based flours, which showed no significant difference (*p* > 0.05, Student’s test). Fat levels were the highest in sunflower seed flour (12.5%), followed by apricot (8.7%) and pumpkin seed flour (5.7%). Whole grain (3.7%) and white wheat flour (2.3%) had significantly lower fat content. Our results, in line with literature data, suggest that variations in the fat content of flours derived from the same oilseed type are most likely due to differences in oil extraction and seed processing methods. The efficiency of oil extraction from seeds and the resulting residual fat content in the press cake vary significantly by method, with cold pressing typically leaving higher fat levels in the residue compared to solvent or supercritical extraction. Sobczak et al. [17] reported that the sunflower and pumpkin press cakes obtained from cold-press oil production contained 31% and 14% fat, respectively, indicating that cold pressing leaves a notable amount of residual oil in the press cake and influences its potential applications. For apricot oil cake, cold pressing yields about 22% residual lipids, far higher than the 6% from advanced methods like supercritical extraction [18].

Apricot (12.0%) and sunflower seed flour (11.3%) exhibited the highest cellulose contents, with statistically significant differences compared to all other flours (*p* < 0.05, ANOVA). While pumpkin seed flour (5.3%) had the lowest cellulose content among the seed flours, it still contained significantly more cellulose than the wheat-based flours (*p* < 0.05, ANOVA). The high cellulose content in apricot and sunflower seed flours reduces their digestibility compared to flours with lower insoluble fiber levels. However, this characteristic enhances their nutritional value, as their incorporation can contribute to increasing fiber intake. Our apricot flour showed cellulose content in line with the fiber content reported for apricot kernels [5]. Mahde and Fayed [19] reported a crude fiber content of 3.4% in apricot kernels. The higher cellulose content observed in apricot flour in this study is likely due to the removal of oil during processing, resulting in altered relative proportions of macronutrients in the final product.

Statistically significant differences in ash content were observed among all samples (*p* < 0.05, ANOVA). The seed flours, especially those obtained from sunflower (9.8%) and pumpkin (8.3%), exhibited significantly higher ash values than the grain-based flours, whole grain wheat (1.5%) and white wheat (0.5%), indicating a richer mineral profile. The obtained results are consistent with previously reported ash contents of 7.3% in sunflower oilcake and 9.9% in pumpkin seed protein preparations [7,16]. These findings highlight sunflower and pumpkin seed residues as rich sources of micronutrients, suitable for use in new food product development.

The moisture content of oil press cake flours (ranging from 8.0% to 8.7%) did not differ significantly from one another (*p* > 0.05, ANOVA). However, the wheat-based flours exhibited significantly higher moisture levels (9.7% to 11.8%) compared to the oil press cake flours (*p* < 0.05, Student’s test). The moisture content values observed in this study are consistent with previously published data, confirming that flours with moisture levels below 12% are considered microbiologically stable and suitable for extended shelf life [7,16].

When evaluated in the context of EFSA’s Dietary Reference Values (DRVs), sunflower and apricot seed flours offer the most balanced macronutrient profiles, while pumpkin seed flour is particularly suitable for protein-enriched formulations or low-carbohydrate diet. In general, oil press cake flours exhibited significantly higher protein and fat contents compared to traditional grain flours, which were predominantly carbohydrate-rich. Pumpkin seed flour was especially protein-dense (65.2%) and extremely low in carbohydrates (6.8%), making it ideal for high-protein applications. Sunflower and apricot seed flours demonstrated a more balanced macronutrient composition, aligning better with EFSA’s recommended energy distribution [20] and providing versatile options for functional food development. In contrast, whole grain, and especially white wheat flour, were dominated by carbohydrates, offering limited nutritional diversity unless fortified or blended with other flours.

### 2.2. Amino Acid Composition of Flours

The quality of dietary proteins relies on their amino acid composition, particularly the content and availability of essential amino acids, which are crucial for overall maintenance of the human body, growth, and reproduction [21,22]. Grain-based flours have been extensively used for centuries due to their global availability and agronomic significance. However, it is important to consider the quality of protein in grains, as they often lack one or more essential amino acids. It is a well-known fact that the lysine content in grains including wheat is generally unsatisfactory [23]. Our study confirms this assertion, given that we measured significantly lower amounts of lysine at 0.51 g/100 g in white wheat flour and 0.67 g/100 g in whole grain wheat flour, versus 1.57–2.54 g/100 g in oil press cake flours (*p* < 0.05, ANOVA) (Table 2). In order to ensure adequate daily intake of protein and essential amino acids, the inclusion of alternative flours, such as those derived from oilseeds, should be prioritized. Aligning with the EAT-Lancet Commission’s emphasis on sourcing protein primarily from plant-based foods for both health and sustainability [24], oil press cake flours provide a valuable and sustainable source of good quality protein. In proper combination with other plant-based proteins like legumes, they can serve as acceptable alternatives to animal protein sources.

Table 2 reveals the results of the amino acid composition of the analyzed flours. The higher total essential amino acid content in oil press cake flours compared with wheat flours (*p* < 0.05, ANOVA) is in agreement with earlier reports [25,26]. The exception was L-methionine, with a higher amount in wheat flour compared to the apricot seed flour sample. However, a lower level of L-methionine in apricot seed flour was also confirmed in a recent study [27]. Pumpkin seed flour had the highest content of all essential amino acids, aligning with its superior protein content (Table 1).

The amino acid proportion in analyzed flours compared to total amino acid content was also analyzed. Among the essential amino acids, leucine was the most abundant across the analyzed flours. Leucine is a branched-chain amino acid that is essential for protein synthesis, tissue regeneration, and metabolism, making it very popular among athletes and fitness trainers. Methionine is present in the smallest relative amount in all samples except pumpkin seed flour, where histidine was found in a minimal percentage consistent to previous studies [26,27].

Glutamate was identified as the dominant non-essential amino acid in all samples, except for white wheat flour, which aligns with previous findings [7,28,29]. Glutamate is one of the most abundant amino acids in the diet and plays several crucial roles in the human body, including nutrition, protein synthesis, metabolism, and cellular signaling. It is essential for the synthesis of important molecules such as glutathione and folate cofactors that contain polyglutamate. The levels of glutamate found in the analyzed samples were as anticipated and desirable. However, in the white flour sample, arginine was the most abundant non-essential amino acid. Arginine and aspartate were also present in a high percentage in all oil press cake flour samples. The high content of arginine in foods is particularly important because it serves as the key precursor for the production of nitric oxide (NO) in the body. Nitric oxide synthases (NOS) use L-arginine as a substrate to produce NO and citrulline. This reaction is vital for various physiological processes, including vasodilation, immune function, and neurotransmission. Wheat flour had a high percentage of proline (11.6% in white wheat flour and 9.8% in whole grain wheat flour), which was not observed in the oil press cakes (3.9% in pumpkin flour, 3.8% in sunflower flour, and 5.2% in apricot seed flour), even though the total amount of proline was greater in the oil press cake flours (Table 2). Cysteine was found in the smallest percentage in all samples.

These compositional data indicate that cold-pressed oil cake flours have the potential to complement wheat flour in blended formulations by compensating for limiting amino acids in cereal proteins and thereby improving the overall amino acid balance of wheat-based products. In this way, the present profiles support the idea that incorporating oil press cake flours as complementary protein sources may enhance the nutritional quality of wheat-containing foods, even though this potential still needs to be confirmed in future technological and nutritional studies.

At the same time, these findings describe compositional potential rather than confirmed protein quality. Because protein digestibility, antinutritional factors, and derived protein quality indices (e.g., PDCAAS, DIAAS) were not determined in this study, the implications for true protein quality and amino acid bioavailability should be interpreted with caution. The proposed improvements in wheat-based formulations should therefore be regarded as preliminary and require confirmation in future work that includes digestibility and bioavailability assessments, as well as technological and sensory trials.

### 2.3. Mineral Content of Flours

Oil press cake flours exhibited a significantly higher content of the analyzed minerals compared to wheat flours (*p* < 0.05, ANOVA), a finding that is consistent with the elevated ash values observed in the corresponding analyses (Table 1). The previous study had similar conclusions; oil press cake flours have been characterized by a higher mineral content compared to wheat flour [26].

The analysis of the macromineral content showed that pumpkin seed flour had the highest potassium content (1916 mg/100 g), followed by sunflower seed flour (1411.6 mg/100 g) and apricot seed flour (1440.9 mg/100 g) (Table 3). As in a previous study [30], a high potassium content relative to sodium was found in oil press cake flour samples. Wheat flours had lower potassium and sodium levels; sodium level in white wheat flour was even below the limit of detection. Sunflower seed flour is noted by the highest calcium content (1937 mg/100 g). It is important to note that the calcium level measured in the sunflower seed flour sample is unusually high compared to results from other studies, such as that by Vasudha et al. [31], which measured 650 mg/100 g sunflower seed cake, while others reported even lower calcium content in sunflower seed flour [32]. Therefore, we must acknowledge that high levels of calcium may partly reflect matrix characteristics or sample heterogeneity, which warrants cautious interpretation of these results.

Microelements such as manganese (12.07 mg/100 g) and copper (2.92 mg/100 g) were also found in the highest amounts in sunflower samples, similar to the results of other studies [7,31]. However, the notably high manganese levels detected in some samples warrant careful consideration due to the neurotoxic potential associated with excessive intake. The Recommended Dietary Intake (RDI) is 2.3 mg/day for adult men and 1.8 mg/day for adult women [33], while the Tolerable Upper Intake Level is set at 11 mg/day, indicating that the manganese concentrations in sunflower and pumpkin seed flours may pose a potential risk. Prolonged exposure (6–24 months) to elevated manganese levels has been linked to manganism, a neurological condition associated with impaired cognition, reduced intellectual performance, and behavioral disturbances [34]. The iron content was the highest in sunflower seed flour (29.83 mg/100 g), followed by pumpkin (14.66 mg/100 g) and apricot seed flour (7.93 mg/100 g), while zinc was found to a greater extent in pumpkin (17.73 mg/100 g), apricot (8.83 mg/100 g), and sunflower seeds (6.82 mg/100 g). The dietary reference intake (DRI) for iron is 8 mg/day for men and 18 mg/day for women, while DRI for zinc is 11 mg/day for men and 8 mg/day for women [35]. In wheat flours, especially in the sample of white wheat flour, the content of these minerals was significantly lower (*p* < 0.05, ANOVA). Although the total selenium concentrations in all samples were modest, the analyzed flours, most notably those derived from apricot and pumpkin seeds, emerge as appreciable dietary sources when evaluated against the recommended daily intake of 55 μg/day, underscoring their potential to enhance selenium status through regular consumption.

While these results point to a promising mineral profile, it is important to note that grains, seeds, and their derived products also contain antinutritional components (e.g., phytic acid, saponins, oxalates, enzyme inhibitors, tannins, and lectins) that can reduce mineral bioaccessibility [36,37]. As antinutritional factors were not measured in this study, the reported mineral levels should be viewed as compositional potential rather than evidence of actual mineral absorption, and future work should examine how processing of oil press cakes can modulate these compounds and their impact on mineral availability.

Overall, it is important to note that mineral composition of these flours can be significantly influenced by factors such as climatic variations, irrigation practices, agricultural methods, soil fertility, and genetic differences. These factors may account for discrepancies observed among these results and previous studies [38].

### 2.4. Total Phenolic Content of Flours

**Figure 1:**
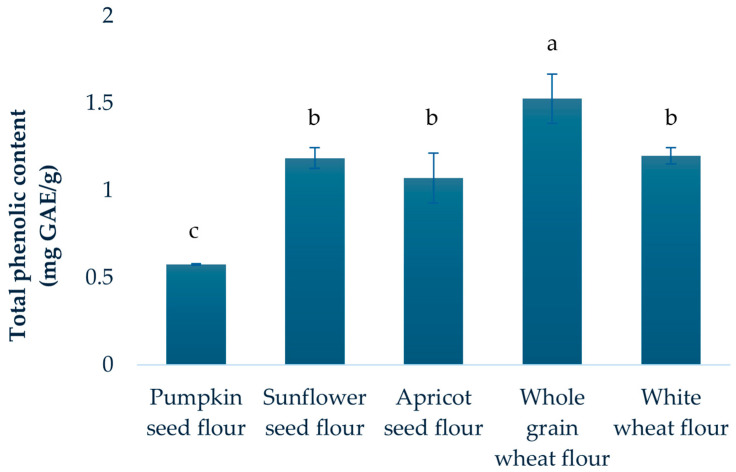
Total phenolic content of analyzed flours. Results are expressed as mean ± standard deviation. a–c: Different letters denote significant differences between means at *p* < 0.05, ANOVA.

The total phenolic content (TPC) of the analyzed flour samples is presented in Figure 1. Whole grain wheat flour showed the highest phenolic content (1.53 mg GAE/g), followed by white wheat flour (1.20 mg GAE/g), sunflower seed flour (1.19 mg GAE/g), and apricot seed flour (1.07 mg GAE/g). Pumpkin seed flour, however, displayed a substantially lower TPC.

When comparing TPC values for all flours to literature data, some variation was expected, given differences in raw materials and experimental conditions [39,40,41,42]. For instance, Kausar et al. [41] reported notable higher total phenolic content in powders derived from whole dry sunflower seeds in contrast to the present results. This was not surprising, considering that our study focused on sunflower seed flour obtained as a by-product of oil extraction. Further, Taha et al. [42] focused on optimization of the extraction of total phenolics from sunflower meal defatted by Soxhlet method and they reported generally higher TPC, which may be attributed to manual sample preparation and the application of advanced extraction techniques. These differences are also consistent with the well-known influence of climate, agricultural practices, and plant variety on phenolic content, as previously observed for apricot kernels [10].

While statistically significant differences were evident among samples, sunflower seed, apricot seed, and white wheat flour constituted a subgroup with no significant differences observed between them (*p* > 0.05, ANOVA). Contrary to expectations, wheat flours demonstrated significantly higher concentrations of polyphenolic compounds compared to the oil press cake flours (*p* < 0.05, Student’s *t*-test). This counterintuitive result may be explained by compositional differences between the flours, including the higher protein content in the oil press cake flours, which can influence phenolic extractability. Previous studies [43,44] have shown that phenolic compounds can strongly interact with proteins to form complexes, which may be soluble or insoluble and mediated by covalent or weaker noncovalent bonds. Such interactions can significantly affect phenolic content and alter the functional and nutritional properties of the flour, potentially lowering protein digestibility [45]. In addition to these biochemical factors, technical aspects may also contribute to the observed pattern. For example, certain phenolics could be lost or redistributed during oil extraction and cake formation in oilseeds. The extraction conditions used in our study may differentially recover free versus bound phenolics from wheat and oilseed matrices, potentially influencing the measured TPC. It should be noted that a detailed exploration of these protein–phenolic interactions was beyond the scope of the present study. Finally, the higher TPC in whole grain wheat flour compared to white wheat flour is in agreement with the findings of Yu et al. [33], and could be explained by the localization of phenolic compounds in cereal grain structures (bran-rich fractions have a high level of bounded polyphenols). Significantly greater phenolic content observed in sunflower oil press cake flour relative to pumpkin oil press cake flour aligns with previous reports [11,34].

### 2.5. Flours Antioxidant Activity

The biological activity of food systems is primarily influenced by their chemical composition. Specifically, the antioxidant response may vary depending on the nature of the reactive species, such as different radicals involved in the reaction. Currently, there is no standardized in vitro method for antioxidant evaluation that fulfills all ideal criteria, such as simplicity, cost-effectiveness, suitability for high-throughput analysis in routine control, the ability to evaluate both hydrophilic and lipophilic antioxidants, etc. To achieve a comprehensive and reliable assessment of the total antioxidant capacity, the application of multiple complementary analytical assays is essential [46]. Therefore, four different methods were employed in this study. The antioxidant potential of sunflower seed flour could not be reliably assessed using the FRAP and CUPRAC assays, as turbidity was observed in the microplate wells. There are multiple possible explanations for this, all lying in the specific chemical composition of sunflower seed flour. Namely, DPPH and TEAC are the most widely employed methods for assessing antioxidant activity in various food and biological matrices, as they are compatible with both hydrophilic and lipophilic compounds and there are no important limitations. On the other hand, the extremely low pH conditions required for the FRAP reaction (pH~3.6) may promote the precipitation of certain proteins [47]. When it comes to CUPRAC assay, it has been reported that some samples require an acidic protein precipitation step prior to the assay to reduce protein-related interferences [48]. Additionally, sunflower seed flour is characterized by relatively high fat content (12.5%, Table 1), substantially greater than that of the other analyzed samples. Similar findings have been reported in previous studies [40]. This elevated fat content may contribute to the observed discrepancies in assay performance. Turbidity encountered in these two assays is the experimental limitation that should be addressed in future work using optimized extraction and clarification approaches.

The results of analyzed antioxidant activities are presented in Table 4. DPPH assay revealed apricot seed flour as the most potent one, followed by sunflower seed flour, whole grain wheat flour, white wheat flour, and pumpkin seed flour. In contrast to the results reported in the previous study [39], our DPPH assay demonstrated a statistically significant difference in antioxidant activity between whole grain and white wheat flour. Similar flour sequence was observed by TEAC assay; apricot has the highest antioxidant activity (5.78 ± 0.92 µmol TE/g), then whole grain wheat flour, sunflower seed flour, white wheat flour, and pumpkin seed flour. Domination of apricot seed flour and whole grain wheat flour was confirmed by FRAP assay. Within the framework of the FRAP assay, thiol groups of proteins do not participate in the redox reaction [49]. Consequently, the antioxidant activity of oil press cake flours may be underestimated, considering the substantial contribution of proteins to the antioxidant potential of high-protein foods [50]. Results obtained using CUPRAC assay were markedly greater than all other performed tests, due to its greater sensitivity to a broad range of compounds, including thiol and lipophilic antioxidants, which are less effectively detected by other methods. Apricot seed flour once again exhibited the highest antioxidant potential; however, white wheat flour also demonstrated considerable antioxidant activity. Oil press cake flours generally displayed higher antioxidant capacities than wheat flours, although these differences lacked statistical significance (*p* > 0.05, Student’s test).

The relatively low antioxidant capacity of pumpkin seed flour has been previously documented [40]. Apricot seed flour consistently exhibited the highest antioxidant activity, irrespective of the applied analytical method. The pronounced antioxidant activity of apricot seed flour may be attributed to the presence of other hydrophilic bioactive compounds with antioxidant potential. Additionally, it is possible that a fraction of lipophilic antioxidants, such as tocopherols and carotenoids, remained bound within the flour matrix and were subsequently extracted, thereby contributing to the observed antioxidant activity. Although the Folin–Ciocalteu assay is non-specific for phenolic compounds [51], the observed lack of correlation between total phenolic content and most antioxidant assays (with the exception of the FRAP assay; r = 0.587, *p* < 0.05, Pearson’s correlation) may, at least in part, result from the presence of these lipophilic antioxidants. Separate analyses of lipophilic antioxidants could provide additional insights, and the lack of such analyses has been identified as both a methodological limitation and a priority for future research.

### 2.6. Study Strengths and Limitations

A key strength of the present study is its focus on the nutritional analysis of flours made from oilseed processing residues, whose valorization can contribute to positive environmental outcomes. Redirecting these by-products from landfills, they may help reduce soil and water contamination while supporting a circular economy. Additionally, incorporating such flours into the food system can lower the demand for conventional flours, thereby decreasing the environmental footprint associated with crop production and processing. While the exact magnitude of these potential benefits cannot be determined, recent studies support their positive environmental impact [52]. By linking nutritional assessment with sustainability, our study underscores oil press cake flours as nutritionally valuable alternatives aligned with the principles of the planetary health diet.

Several limitations should be acknowledged. First, oil press cake flours may contain antinutritional factors such as phytic acid, tannins, and enzyme inhibitors, which can reduce nutrient bioavailability and partly limit the nutritional value of the flours [36,37,53]. Neither the content of these compounds nor potential protein–polyphenol interactions, which are known to influence digestibility and bioactivity [43,44,45,54], were assessed in this study. As a result, the interpretation of the findings is limited. Both aspects represent important areas for further investigation. Furthermore, the relatively small sample size (local manufacturers, specific region) limits the representativeness of the results. Finally, variability arising from growing conditions, agricultural practices, genetic factors, and storage could not be controlled since the samples were obtained post-production, which constrains the generalizability of our findings to other production systems and varieties.

To guide future work, we recommend expanding the sample set, controlling processing conditions, as well as incorporating measurements of key antinutritional compounds and digestibility parameters, such as those required for calculating protein quality indices. These steps would strengthen the nutritional assessment and provide a clearer roadmap for methodological improvements in subsequent studies.

## 3. Materials and Methods

### 3.1. Flour Samples

This study included five types of flour: pumpkin seed, sunflower seed, apricot seed, white wheat, and whole grain wheat flour, with the latter two serving as reference materials. The oil press cake flours were produced by a certified local manufacturer in the North Banat region of Serbia from cold-pressed seed cakes that were conditioned and stored under controlled temperature and humidity. Cakes were milled on a low-speed stone mill and packed in sealed bags before delivery. The wheat flours (white and whole grain) were purchased from a local supermarket. Detailed information on the cold-pressing and milling procedures for the oil press cakes, as well as the labeled nutritional composition of wheat flours, is provided in the Supplementary Material (Figure S1 and Table S2, respectively). All samples were stored in cool, dry conditions until analysis and were analyzed in triplicate.

### 3.2. Determination of Energy Value and Macronutrients Composition

Percentage of moisture, proteins, fats, and ash were determined using the procedures recommended by the Association of Official Analytical Chemists (AOAC) International [55]. The moisture and crude ash content were estimated gravimetrically (AOAC Method 930.04 and AOAC Method 930.05; respectively), the protein (N × 6.25) was determined by the Kjeldahl method (AOAC Method 977.02), and the crude fats by Soxhlet extraction with petroleum ether (boiling point, 40–60 °C), (AOAC Method 930.09). Cellulose content was determined gravimetrically using a selective chemical method to isolate cellulose from other dietary fiber constituents [56]. Total carbohydrates were calculated as the residual difference after subtracting protein, crude ash, moisture, and crude fat content from 100%.

### 3.3. Determination of Amino Acid Composition

Amino acid composition was determined using ion chromatography (IC) with electrochemical detection (silver reference electrode (Ag/AgCl) and gold (Au) working electrode) [57]. Flour samples were previously hydrolyzed; 50 mg of homogenized flour in 300 μL 6M HCl and 0.1% phenol at 110 °C for 1 h. After hydrolysis, samples were cooled to room temperature and dissolved in 25 mL of ultrapure water. Subsequently, prepared samples were filtered through 0.22 μm pore size filter, transferred into a vial and underwent chromatography analyses procedure. The range of calibration curve was 0.5–5 μmol/L for L-cysteine and 1–10 μmol/L for all the other amino acids, with minimal coefficient of correlation 0.95. Limit of quantification for all measured amino acids was 0.01 g/100 g. The results are expressed as mass of amino acids (g) per 100 g of flour sample.

### 3.4. Determination of Mineral Content

The content of minerals was determined using Inductively Coupled Plasma-Mass Spectroscopy (ICP/MS, model 7800, Agilent Technologies, Inc., Arcade, New York, NY, USA) method for detecting metals at very low concentrations [58]. Concentrated nitric acid 65% was used for sample digestion. All dilutions were made with ultrapure water. Calibration standards were prepared using reference standard materials for ICP, which are traceable to a Standard Reference Material (SRM) from the National Institute of Standards and Technology (NIST). These reference materials included certified concentrations for zinc, iron, selenium, manganese, copper, potassium, sodium, and calcium solutions in a 2–3% HNO_3_, with a concentration of 1000 mg/L. The internal standard (rhodium in 2–3% HNO_3_) was used at an equivalent concentration. Limit of quantification for K, Na, and Ca was 10 mg/kg and for Fe, Mn, Zn, Cu, and Se, 0.01 mg/kg. The results are expressed as mg of mineral per 100 g of flour. ICP/MS was found to have good accuracy and repeatability. Detailed sample preparation information and validation parameters are given in Supplementary Files—Figure S2 and Table S2, respectively.

### 3.5. Determination of Bioactive Potential

#### 3.5.1. Flour Extracts Preparation

Extraction procedures were carried out under the following conditions: approximately 2.5 g of each sample was accurately weighed into individual flasks. Subsequently, 25 mL of 80% ethanol was added to each flask. The applied sample/solvent ratio was selected according to the outcomes of a previously conducted optimization procedure [59]. The mixtures were vigorously mixed and subjected to extraction on a shaker for 60 min. Thereafter, the mixtures were centrifuged at 4430× *g* for 20 min using a Hermle Z 206 A centrifuge (Wehingen, Germany). The resulting supernatants were collected and reserved for further analytical procedures.

##### Total Phenolic Content Determination

The total phenolic content was determined using the Folin–Ciocalteu (FC) colorimetric method in a microtiter plate format, as described by Attard et al. [60]. Prior to analysis, the sample extracts were appropriately diluted. Subsequently, 10 μL of diluted extract was mixed with 100 μL of commercially available FC reagent, previously diluted tenfold. Following this, 80 μL of a 7.5% aqueous sodium carbonate (Na_2_CO_3_) solution was added. After the incubation period (60 min at 30 °C), the absorbance of the reaction mixtures was measured at 630 nm using a microplate reader (ELx800 Absorbance Microplate Reader, BIOTEK, Santa Clara, CA, USA) relative to a blank. Quantification of phenolic content was carried out using a calibration curve constructed with gallic acid (0.1 g/L) as a standard. The results are expressed as milligrams of gallic acid equivalents per gram of sample (mg GAE/g).

##### Antioxidant Activity Assessment

The antioxidant activity was investigated using four different colorimetric assays. Absorbance of the reaction mixtures was measured using a microplate reader against a blank. Quantification was carried out using a standard curve prepared with Trolox ((±)-6-hydroxy-2,5,7,8-tetramethylchroman-2-carboxylic acid) and the results are expressed as micromoles of Trolox equivalents per gram of sample (µmol TE/g).

- 2,2-diphenyl-1-picrylhydrazyl (DPPH) method

The antioxidant potential of the extracts was evaluated using the 2,2-diphenyl-1-picrylhydrazyl (DPPH) method, with minor modifications to the procedure described by Norma et al. [61]. The working DPPH solution was freshly prepared by mixing a DPPH solution (1.86 × 10^−4^ mol/L in ethanol) with 0.1 M acetate buffer in a 2:1 (*v*/*v*) ratio. Diluted extract samples (7 µL) were dispensed into the wells of a microtiter plate, followed by the addition of 193 µL of DPPH working solution. The mixture was incubated in the dark at room temperature for 30 min. After incubation, the absorbance was measured at 490 nm.

- Ferric Reducing Antioxidant Power (FRAP) method

The Ferric Reducing Antioxidant Power (FRAP) method was conducted in accordance with the procedure described by Bolanos De La Torre et al. [62], with some modifications. The FRAP working solution was freshly prepared by mixing acetate buffer (pH 3.6), 10 mM TPTZ (2,4,6-tripyridyl-s-triazine) dissolved in 40 mM HCl, and 20 mM FeCl_3_·6H_2_O in a volume ratio of 10:1:1. For the assay, 20 µL of the appropriately diluted extract was added to each well of a microtiter plate, followed immediately by the addition of 280 µL of the FRAP working solution. The reaction mixtures were incubated for 40 min at 37 °C with gentle agitation. Following incubation, the absorbance was measured at 630 nm.

- Trolox Equivalent Antioxidant Capacity (TEAC) method

The Trolox Equivalent Antioxidant Capacity (TEAC) assay was performed according to the method adapted for use with microtiter plates, as described by Pastoriza et al. [63]. Initially, two solutions were prepared: a 7 mM ABTS (2,2′-azinobis(3-ethylbenzothiazoline-6-sulfonic acid)) stock solution and a 2.45 mM potassium persulfate solution, both in 5 mM phosphate buffer. These solutions were mixed in equal volumes and incubated in the dark at room temperature for 12 to 16 h to allow the generation of ABTS^+^• radicals. Prior to the assay, the resulting mixture was diluted with phosphate buffer (~1:80, *v*/*v*) to obtain an absorbance in the range of 0.68 to 0.72 at 734 nm, producing the ABTS working solution. For the assay, 20 µL of the appropriately diluted extract was pipetted into the wells of a microtiter plate, followed by the addition of 280 µL of the ABTS working solution. The reaction mixtures were incubated at 30 °C for exactly 5 min. Absorbance was then measured at 630 nm.

- Cupric ion reducing antioxidant capacity (CUPRAC) method

The cupric ion reducing antioxidant capacity (CUPRAC) assay was conducted following the slightly modified method described by Zengin et al. [64]. For the assay, 67 µL of appropriately diluted sample was added to each well of a microtiter plate, followed by sequential addition of 61 µL of 0.01 M CuCl_2_ solution, 61 µL of 1 M ammonium acetate buffer (pH 7.0), and 61 µL of 7.5 × 10^−3^ M ethanolic neocuproine solution. The reaction mixtures were incubated at room temperature for 30 min with gentle agitation. After incubation, the absorbance was measured at 450 nm.

### 3.6. Statistical Analysis

All analyses were performed in triplicate, and the results are presented as mean values ± standard deviation. The obtained values were processed using SPSS 26.0 software (IBM, New York, NY, USA). Prior to statistical processing, the data were tested for normal distribution applying Shapiro–Wilk test. The difference between samples was determined by a one-way analysis of variance (ANOVA). Given the normal distribution of the data and homogeneity of variances, Tukey’s post hoc test was performed. Comparison between oil press cakes and wheat-based flours was performed by Student’s *t*-test. Pearson’s correlation coefficient was used to check the relationship between the variables. The statistical significance level between the variables was set at *p* < 0.05.

## 4. Conclusions

Rising consumer interest in nutrient-dense, sustainable foods has increased attention on flours derived from oil press cakes as alternatives to conventional cereal flours. In this study, pumpkin, sunflower, and apricot oil cake flours showed higher protein, fiber, and mineral contents, as well as notable antioxidant characteristics, compared with wheat flours, indicating their potential as value-adding ingredients in cereal-based products. Pumpkin seed flour’s exceptionally high protein makes it ideal for protein enrichment, while apricot and sunflower seed flours provide balanced macronutrients suitable for diverse product formulations. Sunflower seed flour’s mineral richness supports micronutrient fortification; apricot seed flour’s antioxidant capacity offers potential in oxidative stress-related applications. However, these conclusions are based on compositional and in vitro data only; sensory properties, processing behavior, antinutritional factors, digestibility, and economic or industrial feasibility were not assessed and may limit practical applications. Future work should therefore integrate technological trials, digestibility and bioavailability assessments, and broader sourcing of raw materials to more fully define the role of oil press cake flours in food formulation.

## Figures and Tables

**Table 1:** Energy and macronutrient composition (per 100 g) of analyzed flours.

Parameter	Pumpkin Seed Flour	Sunflower Seed Flour	Apricot Seed Flour	Whole Grain Wheat Flour	White Wheat Flour
Energy value (kJ)	1439 ± 23 ^b^	1451 ± 33 ^a,b^	1469 ± 31 ^a,b^	1491 ± 20 ^a,b^	1522 ± 25 ^a^
Protein (%)	65.2 ± 2.3 ^a^	34.8 ± 1.6 ^b^	30.3 ± 0.2 ^c^	10.6 ± 0.9 ^d^	9.4 ± 0.5 ^d^
Carbohydrates (%)	6.8 ± 2.4 ^e^	22.9 ± 2.2 ^d^	36.1 ± 2.8 ^c^	70.5 ± 0.6 ^b^	78.2 ± 0.7 ^a^
Fat (%)	5.7 ± 0.7 ^c^	12.5 ± 1.8 ^a^	8.7 ± 0.9 ^b^	3.7 ± 0.5 ^c,d^	2.3 ± 0.6 ^d^
Cellulose (%)	5.3 ± 0.8 ^b^	11.3 ± 0.5 ^a^	12.0 ± 2.4 ^a^	2.0 ± 0.3 ^c^	0.2 ± 0.1 ^c^
Ash (%)	8.3 ± 0.3 ^b^	9.8 ± 0.3 ^a^	4.9 ± 0.1 ^c^	1.5 ± 0.1 ^d^	0.5 ± 0.1 ^e^
Moisture (%)	8.5 ± 0.1 ^c^	8.7 ± 0.1 ^c^	8.0 ± 0.5 ^c^	11.8 ± 0.3 ^a^	9.7 ± 0.2 ^b^

Results are expressed as mean ± standard deviation. a–e: Different letters in superscript within the same row denote significant differences between means at *p* < 0.05, ANOVA.

**Table 2:** Amino acids composition in analyzed flours.

Amino Acids (g/100 g)	Pumpkin Seed Flour	Sunflower Seed Flour	Apricot Seed Flour	Whole Grain Wheat Flour	White Wheat Flour
**L-Lysine**	2.54 ± 0.01 ^a^	1.9 ± 0.04 ^b^	1.57 ± 0.03 ^c^	0.67 ± 0.03 ^d^	0.51 ± 0.04 ^e^
**L-Threonine**	2.38 ± 0.07 ^a^	1.42 ± 0.07 ^b^	1.13 ± 0.08 ^c^	0.43 ± 0.07 ^d^	0.31 ± 0.03 ^d^
**L-Valine**	3.00 ± 0.13 ^a^	1.66 ± 0.03 ^b^	1.25 ± 0.10 ^c^	0.70 ± 0.04 ^d^	0.65 ± 0.05 ^d^
**L-Isoleucine**	2.71 ± 0.06 ^a^	1.50 ± 0.03 ^b^	1.50 ± 0.04 ^b^	0.54 ± 0.08 ^c^	0.55 ± 0.04 ^c^
**L-Leucine**	3.97 ± 0.08 ^a^	2.68 ± 0.06 ^c^	3.27 ± 0.04 ^b^	1.01 ± 0.09 ^d^	0.99 ± 0.03 ^d^
**L-Methionine**	1.62 ± 0.02 ^a^	0.77 ± 0.03 ^b^	0.21 ± 0.03 ^d^	0.30 ± 0.04 ^c^	0.27 ± 0.04 ^c,d^
**L-Histidine**	1.42 ± 0.04 ^a^	0.80 ± 0.11 ^b^	1.01 ± 0.04 ^c^	0.35 ± 0.03 ^d^	0.33 ± 0.01 ^d^
**L-Phenylalanine**	3.13 ± 0.14 ^a^	1.41 ± 0.04 ^c^	1.72 ± 0.03 ^b^	0.62 ± 0.05 ^d^	0.62 ± 0.03 ^d^
L-Proline	2.42 ± 0.05 ^a^	1.31 ± 0.04 ^c^	1.64 ± 0.07 ^b^	1.21 ± 0.03 ^c^	1.18 ± 0.08 ^c^
L-Glycine	3.94 ± 0.06 ^a^	1.23 ± 0.11 ^b^	1.75 ± 0.06 ^c^	0.83 ± 0.07 ^d^	0.69 ± 0.10 ^d^
L-Alanine	3.67 ± 0.08 ^a^	1.58 ± 0.11 ^b^	1.77 ± 0.08 ^b^	0.73 ± 0.09 ^c^	0.45 ± 0.07 ^d^
L-Serine	3.50 ± 0.12 ^a^	1.70 ± 0.11 ^b^	1.55 ± 0.08 ^b^	0.96 ± 0.03 ^c^	0.56 ± 0.05 ^d^
L-Glutamate	11.71 ± 0.25 ^a^	7.26 ± 0.16 ^b^	5.73 ± 0.06 ^c^	1.61 ± 0.04 ^d^	1.18 ± 0.04 ^e^
L-Aspartate	5.11 ± 0.10 ^a^	3.27 ± 0.06 ^b^	3.25 ± 0.05 ^b^	0.47 ± 0.02 ^c^	0.20 ± 0.01 ^d^
L-Cysteine	0.83 ± 0.06 ^a^	0.58 ± 0.03 ^b^	0.15 ± 0.01 ^c^	0.15 ± 0.01 ^c^	0.17 ± 0.02 ^c^
L-Tyrosine	1.76 ± 0.06 ^a^	0.89 ± 0.02 ^b^	0.92 ± 0.03 ^b^	0.22 ± 0.01 ^c^	0.25 ± 0.02 ^c^
L-Arginine	8.02 ± 0.04 ^a^	4.38 ± 0.03 ^b^	3.05 ± 0.05 ^c^	1.56 ± 0.13 ^d^	1.26 ± 0.07 ^e^
Total EAA	20.78 ± 0.77	12.12 ± 0.57	11.65 ± 0.81	4.62 ± 0.21	4.25 ± 0.22
Total NEAA	40.95 ± 3.19	22.21 ± 2.04	19.81 ± 1.54	7.77 ± 0.50	5.95 ± 0.42
Total AA	61.73 ± 2.57	34.33 ± 1.61	31.46 ± 1.30	12.39 ± 0.42	10.2 ± 0.34

Results are expressed as mean ± standard deviation. a–e: Different letters in superscript within the same row denote significant differences between means at *p* < 0.05, ANOVA. EAA—essential amino acids, NEAA—non-essential amino acids, AA—amino acids. Bold—essential amino acids.

**Table 3:** Mineral composition of analyzed flour samples.

Minerals (mg/100 g)	Pumpkin Seed Flour	Sunflower Seed Flour	Apricot Seed Flour	Whole Grain Wheat Flour	White Wheat Flour
K	1916 ± 47.52 ^a^	1411.6 ± 37.53 ^b^	1440.9 ± 52.65 ^b^	304.4 ± 29.05 ^c^	159 ± 11 ^d^
Na	5.29 ± 0.08 ^a^	4.84 ± 0.64 ^a^	2.26 ± 0.29 ^b^	1.20 ± 0.11 ^c^	ND
Ca	111.60 ± 11.65 ^b^	1937.00 ± 25.00 ^a^	212.23 ± 13.10 ^c^	38.48 ± 3.41 ^d^	22.82 ± 3.56 ^d^
Fe	14.66 ± 0.06 ^b^	29.83 ± 0.35 ^a^	7.93 ± 0.95 ^c^	4.14 ± 0.08 ^d^	0.96 ± 0.02 ^e^
Mn	7.37 ± 0.11 ^b^	12.07 ± 2.00 ^a^	2.27 ± 0.09 ^c^	4.05 ± 0.10 ^d^	0.62 ± 0.03 ^e^
Zn	17.73 ± 0.25 ^a^	6.82 ± 0.59 ^b^	8.83 ± 0.11 ^c^	2.66 ± 0.06 ^d^	0.57 ± 0.01 ^e^
Cu	2.60 ± 0.13 ^a^	2.92 ± 0.11 ^a^	1.85 ± 0.03 ^b^	0.44 ± 0.07 ^c^	0.15 ± 0.03 ^d^
Se	0.05 ± 0.01 ^b^	0.04 ± 0.01 ^a,b^	0.07 ± 0.01 ^a^	0.02 ± 0.01 ^c^	0.01 ± 0.01 ^c^

Results are expressed as mean ± standard deviation. a–e: Different letters in superscript within the same row denote significant differences between means at *p* < 0.05, ANOVA. ND—not detectable.

**Table 4:** Antioxidant activity of analyzed flours.

	Pumpkin Seed Flour	Sunflower Seed Flour	Apricot Seed Flour	Whole Grain Wheat Flour	White Wheat Flour
DPPH	1.73 ± 0.35 ^c^	3.47 ± 0.16 ^b^	4.41 ± 0.13 ^a^	2.92 ± 0.25 ^b^	2.15 ± 0.15 ^c^
FRAP	1.93 ± 0.05 ^b^	ND	5.94 ± 0.60 ^a^	5.34 ± 0.13 ^a^	2.40 ± 0.10 ^b^
TEAC	2.52 ± 0.69 ^b^	3.65 ± 0.25 ^b^	5.78 ± 0.92 ^a^	3.72 ± 0.10 ^b^	3.56 ± 0.06 ^b^
CUPRAC	19.08 ± 1.47 ^d^	ND	63.15 ± 3.87 ^a^	36.23 ± 0.96 ^c^	42.54 ± 1.10 ^b^

Results are expressed as mean ± standard deviation (µmol TE/g). a–d: Different letters in superscript within the same row denote significant differences between means at *p* < 0.05, ANOVA. TE—Trolox Equivalents; ND—not detectable.

## Data Availability

The data sets used and/or analyzed during the current study are available from the corresponding author on reasonable request.

## Supplementary Materials

The following supporting information can be downloaded at: https://www.mdpi.com/article/10.3390/molecules30244781/s1, Figure S1: Cold press oil extraction machine; Figure S2: Inductively Coupled Plasma Mass Spectrometry (ICP/MS); Table S1: Nutritional composition (per 100 g) and manufacturer details of wheat-based flours; Table S2: Validation parameters of ICP-MS method used for mineral determination.

## Abbreviations

The following abbreviations are used in this manuscript:

AA	Amino Acids
ANFs	Antinutritional Factors
ANOVA	One-way Analysis of Variance
AOAC	Association of Official Analytical Chemists
CUPRAC	Cupric Ion Reducing Antioxidant Capacity
DPPH	2,2-diphenyl-1-picrylhydrazyl
DRVs	Dietary Reference Values
EAA	Essential Amino Acids
EFSA	European Food Safety Authority
FC	Folin–Ciocalteu
FRAP	Ferric Reducing Antioxidant Power
IC	Ion Chromatography
ICP/MS	Inductively Coupled Plasma-Mass Spectroscopy
ND	Not Detectable
NEAA	Non Essential Amino Acids
NIST	National Institute of Standards and Technology
NO	Nitric Oxide
NOS	Nitric Oxide Synthases
SRM	Standard Reference Material
TE	Trolox Equivalents
TEAC	Trolox Equivalent Antioxidant Capacity
TPC	Total Phenolic Content
