# Supplementary material for: Nutritional and Antioxidant Comparison of Oil Press Cakes and Wheat Flours
Source: Molecules. 2025 Dec 15;30(24):4781. doi: 10.3390/molecules30244781 (PMC12735919; doi:10.3390/molecules30244781)
Supplement: Supplementary file 1 [file molecules-30-04781-s001.zip › molecules-4004398-supplementary.pdf]

Supplementary Files

Supplementary table (Table S1) presents the declared macronutrient content (per 100 g), manufacturer information, and lot numbers of the wheat-based flours included in this study. The oilseed-based flours used in our study are produced by a local manufacturer currently in the development phase. As such, the official nutritional declarations are still under preparation and not yet available on the product labels. We will be able to provide more comprehensive nutritional information once the finalized declarations become available.

Table S1. Nutritional composition (per 100 g) and manufacturer details of wheat-based flours

Nutrient	Whole grain wheat flour*	White wheat flour**
Energy (kJ)	1424	1483
Fat (g)	2.4	1.3
— of which Saturated Fat (g)	0.5	0.6
Carbohydrates (g)	63.3	73.9
— of which Sugars (g)	0.7	0.4
Fiber (g)	8.4	1.8
Protein (g)	11.3	9.5
Salt (g)	< 0.01	< 0.01

* Manufactor: Maxi; lot number: L2410281210302

**Manufactor: Danubius; lot number: L2411085110141

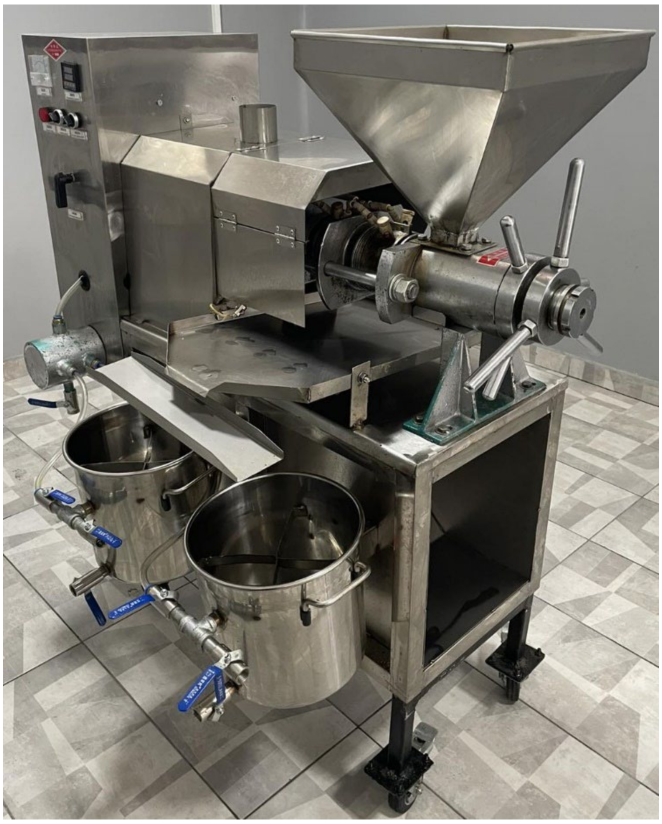

Figure S1: Cold press oil extraction machine

Cold pressing:

The cold oil press operates by slowly pressing seeds through a rotating screw mechanism within a narrow channel. This mechanical pressure extracts oil through a fine mesh without applying additional heat and solvents, thereby preserving the oil's nutritional quality, natural flavor and aroma. The solid residue, known as cake, exits separately and can be used for further processing. In this study, the oilseed cake was subsequently ground into flour, making the extraction process both efficient and sustainable.

Milling and storage procedures:

After cold pressing, the oilseed cakes are conditioned in paper bags and stored in a dark room with a humidity level of 60% and a temperature of 10–18 °C. Proper ventilation is required, and this conditioning process lasts for up to seven days. Milling typically takes place after cold pressing, provided the seed cake humidity is within the ideal range of 6–9%. Milling is performed using a stone mill operating at 80–120 rpm, ensuring the temperature remains below 40 °C. Once milled, the oilseed-based flours are packaged in aluminium foil bags in quantities ranging from 250 to 1000 g. These flours are then stored in separate dark storage rooms, where the humidity should remain below 60% and the temperature should be between 8–15 °C. It is recommended to refrigerate the flour after opening the package.

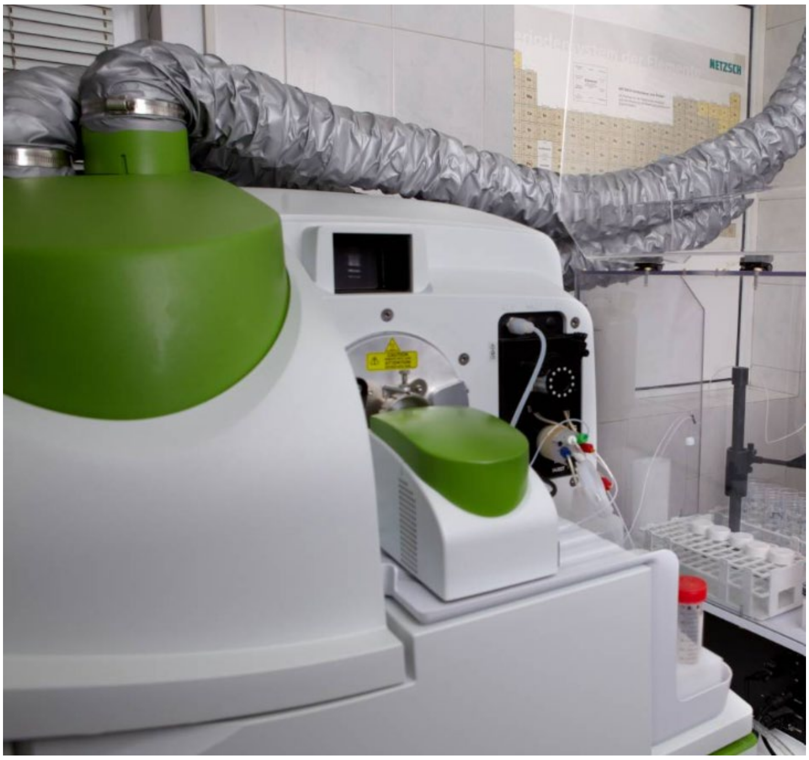

Figure S2: Inductively Coupled Plasma Mass Spectrometry (ICP/MS)

Samples were prepared using microwave (Multiwave 5000, Anton Paar). Homogenised samples (0.5 g) were mixed with 7 ml of HNO_3 (65%) in PTFE (Teflon) cuvette; as a blank 0.5 g of pure water was mixed with 7 ml ccHNO_3 and all undergo microwave digestion procedure. After digestion, all samples, as well as blank, were quantitatively transferred to 50 ml flask, added 0.05 ml of internal standard solution and completed with ultra-pure water, than filtered through membrane filter and placed in cuvette ready for ICP/MS. Measurement was conducted by injecting calibration, blank and all samples solutions into analyser.

Table S2. Validation parameters of ICP-MS method used for mineral determination

Mineral	Linear equation	Linearity R^2	LOD (µg/L)	LOQ (mg/kg)	Repeatability (RSD %)	Accuracy (Recovery %)
K	y=0.0140x+0.9636	0.9997	2.785	10	3.7	97-104
Na	y=0.0260x+0.5595	0.9999	1.382	10	9.9	102-105
Ca	y=0.00071168x+0.0121	0.9999	6.589	10	4.9	101-103
Fe	y=0.004233x + 0.014754	0.9994	2.081	0.01	6.4	84-106
Mn	y=0.1066x+0.3374	0.9998	0.6408	0.01	5.3	95-104
Zn	y=0.0353x+0.1875	0.9998	1.132	0.01	6.9	92-106
Cu	y=0.2882x+0.0969	0.998	0.0461	0.01	9.3	94-108
Se	y=0.0014x+0.00048869	0.9998	0.4581	0.01	9.8	97-105

LOD- Limit of Detection; LOQ- Limit of Quantification; RSD- Relative Standard Deviation

To determine the concentrations of mineral compounds from the ratio of ion intensities calibration curves (x-axis: standard concentrations; y-axis: ratio of ion intensities of mineral signal divided by internal standards signal Rhodium) were constructed. Standards for the calibration curves points were obtained by the series of referent standard dilutions (1000 mg/L) and included five (Cu, Fe, Zn, Mn, Se) or six (Na, K, Ca) different concentrations. The linearity of the calibration curves was evaluated by calculating its coefficient of determination (R^2).

Limit of Detection values were calculated based on measurements from 10 repetitions of blank samples. The stand deviation of these measurements was used in the formula LOD=3*Std. To determine the limit of quantification, a blank was injected 20 times, spiked with a concentration corresponding to the lowest concentration of the calibration curve of each analyte individually. By calculating the standard deviation of the obtained results, according to the SRPS EN 15763 standard, the limit of quantification was determined based on the formula LOQ=6*Std.

Repeatability for each element was determined by spiking samples with the respective analytes, and calculating the relative standard deviation (RSD, %) from replicate measurements. Accuracy of the method was assessed using the standard addition approach (Recovery test); blank samples and samples spiked at one concentration level were repeatedly analyzed over two days, and recovery values were determined.
